# How do low-carbon city policies shape older adults health? Evidence from environmental and mobility pathways in China

**DOI:** 10.3389/fpubh.2025.1649879

**Published:** 2025-08-20

**Authors:** Xiaonan Yue, Yudi Li

**Affiliations:** ^1^College of Foreign Languages and Cultures, Chengdu University of Technology, Chengdu, China; ^2^Graduate School of Advanced Integrated Studies in Human Survivability, Kyoto University, Kyoto, Japan

**Keywords:** low carbon city pilot policy, older adults health, environmental quality, non-motorized travel, spatial accessibility

## Abstract

**Objectives:**

As environmental pollution and population aging become increasingly severe, it is especially important to assess the health co-benefits of climate-related urban policies. This study aims to examine the impact of China’s Low-Carbon City Pilot Program (LCCP) on the health of the older adults. It focuses on potential mechanisms such as improvements in environmental quality and increases in non-motorized transportation.

**Methods:**

This study uses a multi-period Difference-in-Differences (DID) approach to evaluate the health effects of the LCCP. The analysis draws on nationally representative panel data from the China Family Panel Studies (CFPS) between 2012 and 2018. The sample is restricted to individuals aged 60 and above. The main outcome variable is self-rated health, with frequency of medical visits used as a supplementary indicator. The treatment variable is defined based on the official list of low-carbon pilot cities and their launch years. The analysis controls for various individual, intergenerational, and household-level characteristics. To explore possible mechanisms, the study conducts mediation analysis focusing on perceived environmental quality and non-motorized travel behavior. It also includes commuting distance and commuting time as moderating variables to assess the limiting effect of spatial accessibility.

**Results:**

The results show that the LCCP significantly improved the health of older adults. The DID estimates indicate a positive policy effect that remains robust across alternative model specifications and when using other outcome variables such as medical visit frequency. The mediation analysis suggests that improvements in environmental quality—especially in subjective environmental perceptions—and increases in non-motorized travel are key channels for health improvement. In addition, the health benefits of the policy are more pronounced among older adult individuals who face longer commuting distances and times. Subgroup analysis further reveals heterogeneous effects: the policy yields greater health improvements for males, rural residents, and the younger older adults (aged 60–69). Overall, these findings support the proposed hypotheses and highlight both the direct and indirect health benefits of China’s low-carbon urban transition.

**Conclusion:**

This study concludes that the LCCP significantly enhanced older adults health by improving environmental quality and travel behavior. The health effects are stronger for groups with better spatial accessibility and are partially realized through household perceptions and increased non-motorized travel. The results emphasize the importance of integrating environmental policies with age-friendly planning in urban governance to promote healthy aging.

## 1 Introduction

Urban transformation strategies aimed at reducing greenhouse gas emissions are widely recognized for delivering multiple co-benefits, such as improved environmental quality, enhanced well-being, and better public health. Existing studies have shown that low-carbon urban development not only helps mitigate climate change but also generates broader social and health gains (1). In the case of China, its low-carbon transition strategy emphasizes policy integration—particularly through a combination of energy restructuring and pollution control—balancing climate goals with public health outcomes (2). Scholars further argue that achieving carbon neutrality requires not only technological advancement but also substantial changes in social systems and institutions, especially efforts to redesign urban spaces into healthier and more sustainable environments (3). This perspective offers a useful framework for analyzing how environmental improvements induced by policy can affect health outcomes. However, many existing studies have not fully examined how such transformation strategies impact vulnerable groups, especially older adults who are highly exposed to urban environmental stressors.

In the field of urban transportation, researchers have emphasized the health benefits of promoting active travel. Investments in walking, cycling, and public transportation infrastructure can reduce emissions while encouraging physical activity, which improves cardiovascular and metabolic health (4). Policies and urban design that support low-carbon lifestyles can also trigger behavior changes at the community level, generating positive feedback between environment and health (5–7). Yet, much of the current literature focuses on overall behavioral shifts, without identifying which specific behaviors are most responsive to policy and how they translate into measurable health outcomes.

China’s Low-Carbon City Pilot Program (LCCP), launched in 2010, represents a key institutional effort to integrate climate policy into urban governance. Prior studies have found that the LCCP significantly promotes green innovation among firms and strengthens investment in clean technologies and environmental practices (8). Other research shows that the program improves energy efficiency and resource productivity, supporting green economic growth (9). At the same time, the LCCP achieves “dual reductions” in both carbon emissions and traditional pollutants, without undermining economic performance (10).

A growing number of studies have started to explore the LCCP’s health implications. Some have found that improvements in air quality are linked to better health outcomes among the older adults (11, 12), while others associate reduced pollution exposure with lower mortality rates (13). Air pollution is a major risk factor for older adults health in China. Long-term exposure to PM2.5 significantly increases stroke mortality among older adults, particularly in highly polluted northern cities (14). In addition to cardiovascular risks, air pollution harms lung function and cognitive ability, with cumulative exposure accelerating age-related physical decline (15, 16). Addressing these environmental health risks is essential not only for individual well-being, but also for reducing the broader social and economic burdens of an aging population (17, 18). Although the LCCP was primarily designed for environmental governance, it may also generate important health co-benefits by improving living conditions and encouraging healthier behaviors among older adults.

Despite extensive evaluations of the LCCP’s environmental and economic outcomes, its health effects—especially the mechanisms influencing older adults health—remain underexplored. This study contributes to the existing literature in two main ways. First, it integrates environmental mechanisms (such as perceived air quality) and behavioral mechanisms (such as non-motorized travel) to more systematically evaluate how the LCCP affects older adults health, moving beyond a narrow focus on pollution exposure. Second, it introduces spatial moderating variables (such as commuting distance and time) to examine how policy effectiveness varies under different levels of urban accessibility. Together, these approaches broaden our understanding of how climate-oriented urban policies can support healthy aging, and offer a new perspective for assessing the co-benefits of low-carbon development in China and other aging societies.

The remainder of this paper is structured as follows: Section 2 presents the theoretical framework and research hypotheses. Section 3 introduces the data, variable construction, and empirical strategy. Section 4 reports the main findings and discussion. Section 5 concludes with key insights and policy implications.

## 2 Theoretical framework

This study draws on theories from environmental epidemiology, behavioral science, and urban planning to build a framework for understanding how the Low-Carbon City Pilot Program (LCCP) affects older adults health. Environmental epidemiology has shown that residential characteristics—such as neighborhood design, transportation infrastructure, and walkable paths—can significantly influence physical activity, lung function, mental health, and overall quality of life among older adults (19, 20). Behavioral science suggests that features of the built environment, including access to public transportation, commercial services, and green spaces, largely determine older adults travel behavior, especially how often and how long they walk (21). Urban planning research further highlights the positive impacts of urban green spaces on respiratory health, stress reduction, cognitive function, and social engagement (22). In addition, health geography emphasizes the structural relationship between spatial accessibility and health outcomes. It points to commuting distance and time as important moderating variables (23).

Based on these insights, this study focuses on three core mechanisms: the direct health effect, environmental quality improvement, and changes in travel behavior. It also incorporates spatial accessibility as a moderating factor to analyze how the LCCP may influence older adults health through multiple pathways.

### 2.1 Total effects of LCCP on older adults health

China’s Low-Carbon City Pilot Program (LCCP) aims to improve urban infrastructure, reduce greenhouse gas emissions, and promote sustainable practices in energy use, transportation, and land planning. These efforts often lead to overall improvements in the urban environment, including better air quality, higher green coverage, the promotion of energy-efficient buildings, and increased support for low-emission transportation modes (9, 10). Such changes help reduce exposure to environmental stressors like air pollution, urban heat island effects, and noise. These stressors tend to have a stronger impact on older adults due to their greater vulnerability (14, 15).

A large body of environmental health research has shown that improvements in the urban living environment are closely linked to better physical and mental health among older populations (24, 25). Specific benefits include lower risks of respiratory and cardiovascular diseases, improved psychological well-being, and higher life satisfaction. Because older adults are more sensitive to ecological risks due to age-related physiological decline (16), they are likely to benefit more from the wide-ranging improvements brought by the LCCP. Therefore, this study first assesses the overall impact of LCCP implementation on older adults health, without breaking down the specific pathways at this stage.

*H1*: The implementation of the low-carbon city pilot program significantly improves the health of older adults.

### 2.2 Perceived environmental quality as a mediating mechanism

To better understand the pathways through which the LCCP affects older adults health, this study further examines the mediating role of perceived environmental quality. Previous research has shown that environmental quality—especially air pollution—is closely linked to various health risks in older adults, including respiratory diseases, cardiovascular problems, and cognitive disorders (15, 26, 27). Other studies indicate that long-term exposure to polluted environments negatively affects mental health, potentially causing anxiety and reducing life satisfaction (28).

There is growing evidence that the LCCP has significantly improved environmental indicators such as air quality and green space availability (10, 29). These objective improvements are often accompanied by changes in subjective environmental perceptions, which can also independently affect health outcomes. Some studies find that improved perceptions of air cleanliness, noise control, and overall environmental quality are significantly associated with lower depression levels and higher quality of life among the older adults (30, 31). Thus, perceived environmental quality not only reflects the physical condition of the environment but also serves as an important psychological pathway through which policy can influence health.

*H2*: The LCCP improves older adults health in part through enhanced perceptions of environmental quality.

### 2.3 Non-motorized travel as a mediating mechanism

Beyond environmental improvements, the LCCP also aims to reshape urban mobility patterns by encouraging low-carbon transportation, such as walking and cycling. Policy measures often include investments in pedestrian-friendly infrastructure, bike lanes, and safer street designs, which are especially important for older adults with declining physical function (32, 33). These infrastructure upgrades help create an environment that supports daily mobility and promotes physical independence in later life.

A large body of research has shown that non-motorized travel has significant health benefits for the older adults. It can improve cardiovascular function, lower the risk of chronic diseases such as hypertension and diabetes, enhance balance and mobility, and reduce the risk of falls (34–37). Walking and cycling are also associated with lower levels of depression and anxiety, and they offer more opportunities for social interaction and cognitive stimulation (30, 38). As urban travel conditions improve under the LCCP, older adults may shift from passive transportation modes, like private cars, to active modes such as walking or cycling. This behavioral shift may be a key pathway through which low-carbon urban policies promote healthy aging. Therefore, this study tests whether increased non-motorized travel mediates the effect of the LCCP on older adults health.

*H3*: The LCCP improves older adults health in part through increased non-motorized travel.

### 2.4 Spatial accessibility as a moderating factor

Although the LCCP may improve environmental and behavioral conditions, its health benefits are unlikely to be equally distributed across all older adults. One key factor contributing to such inequality is spatial accessibility—that is, the distance and time older individuals need to reach basic services and community facilities. When accessibility is limited, opportunities for physical activity, social interaction, and access to health services are reduced, weakening the potential health benefits of policy improvements.

Research in gerontology and urban studies has shown that longer travel distances and commuting times discourage older adults from participating in physical and social activities. This leads to more sedentary lifestyles, greater social isolation, and worsening health outcomes (39–41). The impact is especially severe for those with limited mobility or poor transportation options. In contrast, older adults living closer to parks, clinics, or community centers are more likely to engage in routine health-promoting activities (42–44). Therefore, spatial accessibility may determine the extent to which LCCP-related improvements translate into actual health gains for older adult residents. This study defines spatial accessibility using commuting distance and commuting time, and examines how these factors moderate the policy’s health effects.

*H4*: The positive impact of the LCCP on older adults health is moderated by commuting distance and time, with stronger health benefits observed among those with shorter commutes.

## 3 Research methods

### 3.1 Data source

This study uses data from four waves (2012, 2014, 2016, and 2018) of the China Family Panel Studies (CFPS). The CFPS is a nationally representative, longitudinal survey conducted by the Institute of Social Science Survey (ISSS) at Peking University. It aims to capture comprehensive information on the socioeconomic conditions of Chinese residents. The survey covers 25 provinces, 162 counties or districts, and 635 villages or communities, representing about 95% of China’s population. In the 2010 baseline survey, household and individual response rates were 81.3 and 84.1%, respectively (45). Although the CFPS also includes data from 2010 and 2020, these waves are excluded from the current study due to significant differences in travel-related question design and a heavier reliance on proxy responses in older adults health sections.

This study defines individuals aged 60 and above as “older adults.” After data cleaning and sample selection, a total of 23,567 raw observations were initially obtained. The processing steps include: (1) Removing individuals who do not meet the age criteria; (2) Dropping observations with missing values in key variables, including self-rated health, the policy treatment variable, and core control variables such as age, gender, household registration (hukou), and income; (3) Deleting or trimming extreme values in commuting time (e.g., one-way commutes over 240 min) and income, using the 1st and 99th percentiles to reduce the influence of outliers.

The final dataset is a balanced panel with 17,382 valid observations, covering both the treatment and control groups across four survey waves. This provides a solid foundation for applying the difference-in-differences (DID) method in the following analysis.

### 3.2 Measurement strategy

#### 3.2.1 Dependent variable

The dependent variable in this study is the health status of older adult individuals in China. To ensure comparability across different age groups among the older adults, this study uses a proxy-reported health assessment question from the CFPS: “How would you rate the respondent’s overall health?” This question is answered by a household member and uses a 7-point Likert scale, ranging from 1 (very poor) to 7 (very good). A higher score indicates better health status. This variable is treated as continuous, which is a common practice in the existing literature.

To improve the robustness of health measurement, this study also includes the number of medical visits in the past year as a supplementary indicator. Frequent medical visits often reflect poorer health conditions and can serve as an objective complement to subjective self-rated health.

#### 3.2.2 Independent variable

The key independent variable is a binary indicator that identifies whether a county or city was included in the Low-Carbon City Pilot (LCCP) program. According to the National Development and Reform Commission (NDRC), the policy was implemented in three batches: July 19, 2010 (first batch), November 26, 2012 (second batch), and January 7, 2017 (third batch). The first batch included 5 provinces and 8 cities. The second batch added 1 province and 28 cities. The third batch included an additional 45 cities (or districts/counties). In cases where only a province was designated as a pilot region, the official documents were used to identify the specific cities included in the program.

Considering that the second batch was announced at the end of 2012 and that policy implementation typically involves a time lag, this study treats 2013 as the actual start year for the second batch. Accordingly, the first batch is considered effective from 2010, the second from 2013, and the third from 2017. Cities that were not included in any batch serve as the control group. A full list of pilot cities and their corresponding start years is provided in Supplementary Table A1.

#### 3.2.3 Mechanism variables

This study proposes two potential mechanisms through which the Low-Carbon City Pilot Program (LCCP) may influence older adults health. The first mechanism is the improvement in perceived environmental quality. This variable is based on the CFPS question: “How serious do you think environmental problems are in China?” Respondents rate the severity on a scale from 0 (not serious at all) to 10 (very serious). For better interpretability, this variable is reverse-coded, such that higher values indicate better perceived environmental quality (0 = “very poor,” 10 = “very good”). To enhance robustness, this study also introduces objective environmental indicators at the city level—such as emissions of sulfur dioxide and particulate matter—to validate the reliability of this mechanism.

The second mechanism is a behavioral adaptation pathway, measured by the increase in non-motorized travel. This study defines non-motorized travel as walking or cycling when traveling to an activity center. The variable is derived from the CFPS question: “What is the most commonly used mode of transportation from your home to the activity center?” Respondents choose from seven options: (1) walking; (2) bicycle/tricycle; (3) electric bike/electric tricycle/motorbike; (4) bus/private car; (5) subway; (6) taxi; (7) shuttle provided by the activity center. A binary variable is constructed: it takes the value of 1 if the respondent selects walking or bicycle/tricycle, and 0 otherwise. This definition aligns with the commonly used concept of “active travel” in the literature.

#### 3.2.4 Moderating variables

This study introduces two moderating variables—commuting distance and commuting time—to examine whether mobility constraints affect the health impact of the LCCP.

The “commuting distance” variable comes from the CFPS question: “How far is your home from the activity location?” In the 2012 and 2014 surveys, respondents chose from four categories: (1) less than 1 km; (2) 1–2 km; (3) 2–5 km; (4) more than 5 km. In 2016 and 2018, they reported a specific value (0–100 km). To ensure consistency across waves, this variable is recoded into a binary format. Because the first three categories accounted for only 15% of the total sample, we merged them into a “short commute” group (coded as 0). The fourth category and all values above 5 km are coded as “long commute” (coded as 1).

The “commuting time” variable is based on the CFPS question: “How long does it usually take you to reach the activity location, one way?” Respondents report the time in minutes (ranging from 0 to 240 min). The variable is then split at the median value of 10 min: 10 min or less is coded as “short commute time” (0), and more than 10 min as “long commute time” (1). For robustness checks, this study also uses the mean commuting time of 14 min as an alternative threshold and conducts subgroup regression analysis to test the moderating effect of commuting distance and time on the policy’s health impact.

#### 3.2.5 Control variables

Older adults health can be influenced by various factors at the individual, intergenerational, and household levels (46, 47). Therefore, this study includes control variables across three dimensions: individual characteristics, family member characteristics, and household-level factors.

At the individual level, the controls include age, gender, household registration status (hukou), nighttime sleep duration, and whether the respondent seeks medical care when ill (i.e., treatment-seeking behavior). These variables are widely used in public health research to capture demographic and behavioral health factors (48). For example, sleep duration significantly affects cognitive function and immune response (49, 50), while treatment-seeking behavior reflects health awareness and illness severity (51).

At the family member level, the study controls for the age, years of education, and smoking status of spouses and adult children. These factors reflect the overall family environment. Higher education levels among family members often indicate better socioeconomic status, which can lead to improved living conditions, better access to health information, and more informed medical decisions (52–54). Family members who smoke may expose the older adults to second-hand smoke, which harms their health (55).

At the household level, this study includes the log of last year’s household net income as a control variable. Economic status directly influences access to healthcare, nutrition, and environmental quality—key determinants of older adults health (46, 56). Using the logarithmic form reduces the influence of outliers and better captures relative income differences (57). Omitting income as a control could bias estimates of the LCCP’s health effects.

Definitions of all variables are summarized in Table 1.

**Table 1:** Variable definitions.

Variable category	Variable name	Definition
Dependent variable	Older adults health	Self-rated health of older adults individuals, ranging from 1 (very poor) to 7 (excellent).
Dependent variable	Hospital visit frequency	Number of hospital visits made by the older adults in the past year.
Independent variable	Low-carbon city pilot (DID)	Whether the city was a low-carbon pilot area in that year (1 = yes, 0 = no).
Mechanism variables	Spouse’s perceived environmental quality	Perceived environmental quality rated from 0 (very poor) to 10 (excellent), reported by the spouse.
Mechanism variables	Son’s perceived environmental quality	Perceived environmental quality rated from 0 (very poor) to 10 (excellent), reported by the son.
Mechanism variables	Older adults non-motorized travel	Whether the older adults usually walk or ride a bicycle/tricycle to the activity center (1 = yes, 0 = no).
Moderating variables	Commuting distance	1 = ≥5 km (long distance), 0 = <5 km (short distance) from residence to activity center.
Moderating variables	Commuting time	1 = above median (long time), 0 = below median (short time) for one-way commute to activity center.
Control variables	Older adults age	Age of the older adult individual.
Control variables	Older adults gender	Gender (1 = male, 0 = female).
Control variables	Urban residency (hukou)	Household registration type (1 = urban, 0 = rural).
Control variables	Sleep duration	Average nightly sleep duration.
Control variables	Seeks medical care when ill	Whether the older adults seek medical treatment when unwell (1 = yes, 0 = no).
Control variables	Living at home	Whether the older adults reside at home (1 = yes, 0 = no).
Control variables	Spouse’s age	Age of the older adults individual’s spouse.
Control variables	Son’s age	Age of the older adults individual’s son.
Control variables	Spouse’s education	Educational level (1 = illiterate to 8 = doctoral degree).
Control variables	Son’s education	Educational level (1 = illiterate to 8 = doctoral degree).
Control variables	Spouse’s smoking	Number of cigarettes smoked daily by the spouse.
Control variables	Son’s smoking	Number of cigarettes smoked daily by the son.
Control variables	Log family net income	Total net household income in the past year (log-transformed).

### 3.3 Empirical strategy

#### 3.3.1 The impact of low-carbon city pilot policies on the health of the older adults

This study investigates the effect of low-carbon city pilot policies on older adults health and explores potential mechanisms. A difference-in-differences (DID) approach is employed to evaluate the causal impact of non-random policy interventions. By comparing changes in outcomes between the treatment and control groups before and after the implementation of the policy, this method enables us to isolate the net policy effect.

Given that the rollout of low-carbon city pilots occurred in three batches across different years, we first adopt a time-varying DID model as follows:


(1) $$\text{Elderly Healt}h_{\mathrm{ict}}=\alpha_{0}+\alpha_{1}\mathrm{DI}D_{\mathrm{ct}}+\alpha_{2}X_{\mathrm{ict}}+\mu_{c}+\theta_{t}+\varepsilon_{\mathrm{ict}}$$

In Equation 1, *i* indexes individuals, *c* denotes counties, and *t* represents the survey year. The dependent variable *ElderlyHealth*_*ic*t_ refers to the self-rated health status of individual *i* in county *c* during year *t.* The core independent variable *DIDct* indicates whether county *c* was part of the low-carbon pilot program in year *t,* defined as the interaction between two binary indicators: *Treatc* (1 if county *c* belongs to the treatment group, 0 otherwise) and *Post_t_* (1 if year *t* is post-policy implementation, 0 otherwise).

The control vector *X_ict_* individual-level characteristics such as age, gender, household registration (hukou), nighttime sleep duration, whether the respondent resides at home, and whether they seek medical treatment when ill; intergenerational characteristics such as the age and education of the spouse and children, and their smoking habits; and the logarithm of household net income in the previous year. *μ_c_* denotes county fixed effects, *θ_t_* captures year fixed effects, and *ε_ict_* is the error term.

To further assess the impact of the low-carbon city pilot program on healthcare utilization among the older adults, this study adopts the same difference-in-differences framework to examine changes in hospital visit frequency. Specifically, the following model is employed:


(1’) $$\text{Hospital Visi}t_{\mathrm{ict}}=\alpha_{0}+\alpha_{1}\mathrm{DI}D_{\mathrm{ct}}+\alpha_{2}X_{\mathrm{ict}}+\mu_{c}+\theta_{t}+\varepsilon_{\mathrm{ict}}$$

In Equation (1’), *Hospital Visit*_*ic*t_ denotes the number of hospital visits reported by older adults individual *i* in county *c* during year *t*. The variable *DID_ct_* represents the policy treatment indicator, and *X_ict_* includes the same set of control variables used in Equation 1, such as individual, intergenerational, and household characteristics. *μ_c_* and *θ_t_* epresent county and year fixed effects, respectively, and *ε_ict_* is the error term. Ordinary Least Squares (OLS) estimation is applied to this specification, allowing us to compare the effect magnitude with the primary outcome of self-reported health.

#### 3.3.2 Potential influence mechanisms

This study further explores the mechanisms underlying the benchmark regression results by applying the traditional mediation analysis method, namely the stepwise regression approach. Two potential pathways are considered: perceived environmental quality and non-motorized transportation usage. The model specifications are as follows:


(2) $$\text{Path}\;a:M_{\mathrm{ict}}=\beta_{0}+\beta_{1}\mathrm{DI}D_{\mathrm{ct}}+\beta_{2}X_{\mathrm{ict}}+\mu_{c}+\theta_{t}+\varepsilon_{\mathrm{ict}}$$

(3) $$\text{Path}\;b:\text{Elderly Healt}h_{\mathrm{ict}}=\delta_{0}+\delta_{1}M_{\mathrm{ict}}+\delta_{2}X_{\mathrm{ict}}+\mu_{c}+\theta_{t}+\varepsilon_{\mathrm{ict}}$$

(4) $$\begin{array}{l} \text{Path}\;c:\text{Elderly Healt}h_{\mathrm{ict}}=\gamma_{0}+\gamma_{1}\mathrm{DI}D_{\mathrm{ct}}+\gamma_{2}M_{\mathrm{ict}}\\ +\gamma_{3}X_{\mathrm{ict}}+\mu_{c}+\theta_{t}+\varepsilon_{\mathrm{ict}} \end{array}$$

In these equations, *M_ict_* denotes the mediating variables, including the perceived environmental quality (reported by spouses or children) and non-motorized transportation usage by the older adults. Equation 2 examines the effect of the low-carbon city pilot policy on the mediators; Equation 3 assesses the impact of mediators on older adults health; and Equation 4 estimates the total effect of the pilot policy while controlling for mediators.

All other variable definitions and fixed effects follow Equation 1. If the coefficient *α_1_* in Equation 1 is significant, and both *β_1_* and *δ_1_* are significant, with the absolute value of *γ_1_* in Equation 4 reduced compared to *α_1_*, it provides evidence of a mediation effect.

#### 3.3.3 Dynamic DID model

To examine the dynamic effects of the low-carbon city pilot policy over time, we estimate the following event-study specification:


(5) $$\begin{array}{l} \text{Elderly Healt}h_{\mathrm{ict}}=\rho_{0}+\sum_{k\neq t_{0}}\rho_{k}({\text{Treat}}_{c}\times {\text{Year}}_{k})\\ +\rho_{1}X_{\mathrm{ict}}+\mu_{c}+\theta_{t}+\varepsilon_{\mathrm{ict}} \end{array}$$

In Equation 5, *i* denotes the individual, *c* denotes the county, and *t* denotes the survey year (2012, 2014, 2016, or 2018). *Treat_c_* is a binary indicator equal to 1 if county *c* belongs to a low-carbon pilot area, and *Year_k_* represents year-specific dummy variables. The base year *t_0_* is excluded to avoid perfect multicollinearity and serves as the reference point for estimating the policy’s dynamic effects. All variable definitions, control variables, and fixed effects remain consistent with Equation 1.

## 4 Research results

### 4.1 Sample description

Table 2 reports the descriptive statistics for the full sample. About 80% of the older adult individuals in the sample live in urban areas, and the proportion of males is slightly higher than that of females. The average self-rated health score is 5.633 on a 1–7 scale, with a median of 6, indicating that most older adults people perceive their health as good or very good. The average nighttime sleep duration is 9.18 h, which falls within the recommended range for older adults.

**Table 2:** Sample characteristics.

Variable name	Obs	Mean	SD	Min	Median	Max
Older adults health	17,382	5.633	1.089	1.0	6.0	7.0
Hospital visit frequency	17,382	1.762	3.529	0.0	0.0	122.0
Treatment indicator (DID)	17,382	0.311	0.463	0.0	0.0	1.0
Spouse’s perception of environmental quality	13,221	3.167	2.632	0.0	3.0	10.0
Son’s perception of environmental quality	14,216	3.453	2.574	0.0	3.0	10.0
Older adults non-motorized travel	13,971	0.634	0.482	0.0	1.0	1.0
Commuting distance	17,370	0.203	0.402	0.0	0.0	1.0
Commuting time	17,382	0.712	0.453	0.0	1.0	1.0
Older adults age	17,382	69.351	3.429	60.0	68.0	79.0
Older adults gender	17,382	0.521	0.5	0.0	1.0	1.0
Urban residency (hukou)	17,378	0.205	0.404	0.0	0.0	1.0
Sleep duration	17,298	9.178	1.017	5.0	9.0	15.0
Seeks medical care when ill	17,314	0.627	0.484	0.0	1.0	1.0
Living at home	17,162	0.825	0.38	0.0	1.0	1.0
Spouse’s age	17,382	37.8	6.151	20.0	38.0	65.0
Son’s age	17,382	35.844	5.995	20.0	36.0	64.0
Spouse’s education	17,295	2.917	1.161	1.0	3.0	8.0
Son’s education	17,301	2.674	1.214	1.0	3.0	8.0
Spouse’s smoking	17,382	7.225	9.729	0.0	0.0	40.0
Son’s smoking	17,382	0.086	1.097	0.0	0.0	20.0
Log family net income	16,583	10.558	1.183	0.0	10.72	16.248

Regarding health service utilization, the average number of hospitalizations in the past year is 1.762. However, the distribution is highly skewed, with many older adult individuals not having been hospitalized at all, reflecting substantial variation in medical needs within the group.

For the key explanatory variable, the mean value of the treatment indicator (DID) is 0.311, meaning that about 31.1% of the sample resides in low-carbon pilot cities. This distribution provides a solid basis for causal identification. As shown in the regression results in Section 4.2, there is a statistically significant association between treatment status and older adults health, supporting the hypothesis that older adults residents in pilot cities report better health outcomes.

In terms of mechanism variables, the average perceived environmental quality reported by spouses and children is 3.167 and 3.453, respectively (on a 0–10 scale). The mean value of the non-motorized travel variable is 0.634, indicating that more than half of the older adults walk or bike to activity centers. Descriptive trends suggest that older adults people in pilot cities tend to report higher perceived environmental quality and are more likely to use non-motorized transport, hinting at possible cognitive and behavioral changes influenced by the policy.

As for moderating variables, around 20.3% of the older adults have commuting distances greater than 5 kilometers, and 71.2% have commuting times longer than 10 min. Preliminary patterns show that individuals with shorter commuting distances or times tend to report better health and higher physical activity levels, while those facing heavier commuting burdens have poorer health and lower community participation. These findings provide initial support for the idea that spatial accessibility may moderate the health effects of the policy—a hypothesis that will be tested further in the regression analysis.

### 4.2 The impact of low-carbon city pilot projects on the health of the older adults

We primarily focus on the health status of older adults individuals aged 60–75. Table 3 reports the DID regression results estimating the impact of the low-carbon city pilot program on older adults health. Column 1 presents univariate results, Column 2 includes individual, parental, and household controls, and Column 3 adds year and county fixed effects.

**Table 3:** Impact of low-carbon city pilot program on the health of the older adults aged 60–75.

Variables	Health (1)	Health (2)	Health (3)
DID	0.173*** (9.702)	0.122*** (6.608)	0.304** (2.108)
Older adults age		0.004 (1.209)	0.006 (1.507)
Older adults gender		0.007 (0.423)	0.013 (0.781)
Urban residency (hukou)		0.052** (2.090)	0.105*** (2.734)
Sleep duration		−0.005 (−0.569)	−0.019* (−1.705)
Seeks medical care when ill		−0.053*** (−2.996)	−0.033 (−1.329)
Living at home		0.081*** (3.504)	0.082** (2.177)
Spouse’s age		−0.011*** (−3.885)	−0.007** (−2.023)
Son’s age		0.009*** (3.311)	0.004 (1.164)
Spouse’s education		0.020** (2.165)	0.034*** (3.043)
Son’s education		0.095*** (9.957)	0.075*** (6.286)
Spouse’s smoking		0.005*** (5.827)	0.005*** (4.012)
Son’s smoking		−0.001 (−0.133)	−0.013 (−1.602)
Log family net income		0.075*** (9.950)	0.044*** (3.878)
County fixed effects	No	No	Yes
Year fixed effects	No	No	Yes
Observations (*N*)	17,382	16,080	15,829
Adj. R^2^	0.005	0.041	0.180

*p < 0.1, **p < 0.05, ***p < 0.01. *T*-values are reported in parentheses, calculated using robust standard errors clustered at the county level.

As shown in Table 3, the DID coefficients across all columns are significantly positive, suggesting that China’s low-carbon city pilot policy has meaningfully enhanced older adults health. Specifically, the DID coefficient in Column 3 is 0.304, indicating a 0.304-point increase in older adults health attributable to the policy intervention. Therefore, Research Hypothesis 1 is validated.

#### 4.2.1 Alternative specifications and outcome indicators

Table 4 presents the results of robustness checks. Column 1 reports the regression results for a restricted subsample of older adult individuals aged 70–75, confirming that the main findings are robust across different age groups. Column 2 presents the results for the frequency of hospital visits in the past year, which serves as an alternative health outcome indicator. The DID coefficient remains statistically significant and negative, indicating that the low-carbon city pilot program reduces the number of hospital visits by the older adults. This finding supports the conclusion that the program effectively improves older adults health.

**Table 4:** Robustness tests.

Variables	Older adults health (aged 60–75)	Hospital visit frequency
DID	0.302** (2.089)	−0.330* (−1.878)
Controls	Yes	Yes
County FE	Yes	Yes
Year FE	Yes	Yes
*N*	15,897	15,829
Adj. R^2^	0.180	0.139

*p < 0.1, **p < 0.05, *** *p* < 0.01. *T*-values are reported in parentheses, based on robust standard errors clustered at the county level. All regressions include county fixed effects, year fixed effects, and control variables related to the older adults, their family members, and household characteristics.

All regressions in Table 4 control for individual, parental, and household-level covariates, as well as county and year fixed effects. The Adjusted R^2^ values of 0.180 and 0.139 suggest reasonable explanatory power of the models. These results confirm the robustness of our main findings and further validate Research Hypothesis 1.

#### 4.2.2 Addressing endogeneity

This study addresses two major endogeneity concerns. The first pertains to the non-random selection of low-carbon pilot cities. These cities were initially nominated by local governments and subsequently approved by the National Development and Reform Commission (NDRC) after a comprehensive review of their specific characteristics. Many pilot cities are economically advanced, rich in natural resources, and possess strong industrial foundations. These inherent advantages may increase both the likelihood of being selected and the health outcomes of the local older adult population, leading to selection bias.

To address potential endogeneity, we first validated the parallel trend assumption. Supplementary Table A2 supports the parallel trend assumption, while Supplementary Table A3 shows that the positive policy effect is consistent across eastern, central, and western regions. To further mitigate potential selection bias in the designation of pilot cities, we employ a Propensity Score Matching combined with Difference-in-Differences (PSM-DID) approach. The PSM-DID results, under effective matching conditions, show that the DID coefficient remains significantly positive and highly consistent with the baseline estimates, confirming the robustness of the findings (see Supplementary Tables A4, A5).

In addition, we adopt an instrumental variables (IV) strategy using a two-stage least squares (2SLS) regression to further address endogeneity. Specifically, we employ two instrumental variables: terrain ruggedness and urban green space area. These variables are selected based on their theoretical relevance and presumed exogeneity. Terrain ruggedness, shaped by natural topography, influences urban density and economic development patterns, making cities with flatter terrain more likely to be chosen as pilot cities. In contrast, urban green space area reflects local governments’ planning decisions and environmental priorities, which are shaped more by geographic and administrative factors than by residents’ health status. Thus, both instruments are plausibly correlated with the policy selection process while remaining exogenous to unobserved determinants of older adults health.

To ensure robustness, each instrument is interacted with the post-policy dummy (Postₜ) to construct the IV for the interaction term. The first-stage regression results (see Supplementary Table A6) demonstrate that both instruments are strongly correlated with the endogenous regressor, with *F*-statistics well above the critical threshold, suggesting no weak instrument problem. The under-identification test rejects the null hypothesis of instrument irrelevance at the 1% level, while the Hansen *J* test supports the joint validity of the instruments. The second-stage results consistently confirm that the low-carbon city pilot program significantly improves older adults health outcomes, reinforcing the baseline findings.

The second source of potential endogeneity arises from omitted variables, such as unobservable concurrent policy shocks or local development factors that may confound the estimated effects. To address this, we conduct placebo tests by randomly assigning treatment status across counties and re-estimating the model. The distribution of the placebo estimates centers around zero, while the actual DID estimate lies in the far-right tail. This suggests that the observed effects are unlikely to be driven by random chance or omitted variables.

In summary, after addressing the key endogeneity concerns—including selection bias and omitted variable bias—our findings remain robust and confirm the causal relationship between low-carbon pilot programs and improved older adults health.

#### 4.2.3 Dynamic impact of low-carbon city pilot policies on the health of the older adults

Table 5 shows the dynamic effects of the low-carbon city pilot policies on older adults health. The positive impact of these policies started to become significant after 2014. This may be due to a time lag between the implementation of the policies and their actual influence on health outcomes. Improvements in air quality and the promotion of green transportation—key goals of the pilot program—often take time to bring about noticeable changes in health, especially among the older adults. As a result, the health benefits of the program appear gradually rather than immediately.

**Table 5:** Dynamic impact of low-carbon city pilot program on the health of the older adults.

Variable	Coefficient	*t*-value
DID2012	0.245	(1.404)
DID2014	0.198	(1.254)
DID2016	0.467***	(3.202)
DID2018	0.310*	(1.793)
Controls	Yes (including spouse, child, and family-level covariates)	
County FE	Yes	Yes
Year FE	Yes	Yes
*N*	15,892	
Adj. R^2^	0.182	

*p < 0.1, **p < 0.05, ***p < 0.01. Robust *t*-values are in parentheses. Clustered standard errors at the county level.

#### 4.2.4 Environmental quality mechanism

Table 6 reports the empirical results for the environmental quality mechanism. Panel A presents findings from the perspective of spouses, while Panel B displays results from the perspective of the older adults themselves. Column (1) shows that the pilot policy significantly improved perceived environmental quality among spouses. Column (2) indicates that higher perceived environmental quality among spouses positively affected older adults health. In Column (3), after controlling for spouse-perceived environmental quality, the DID coefficient remains significantly positive but becomes smaller than in the baseline model, suggesting that part of the policy’s health impact operates through improved environmental quality.

**Table 6:** The potential mechanism of the impact of low-carbon city pilot program on the health of the older adults—by the improvement of environmental quality.

Panel A. Spouse’s perspective	(1)	(2)	(3)
	Path a	Path b	Path c
DID	Spouse’s perception of environmental quality	Older adults health	Older adults health
	0.317* (1.880)		0.290** (2.008)
Spouse’s perception of environmental quality		0.013*** (2.746)	0.013*** (2.702)
*N*	13,024	13,024	13,024
Adj. R^2^	0.098	0.184	0.186

Panel B. Children’s perspective	(4)	(5)	(6)
	Path a	Path b	Path c
DID	Children’s perception of environmental quality	Older adults health	Older adults health
	0.020 (0.136)		0.366*** (2.624)
Children’s perception of environmental quality		0.007* (1.772)	0.007* (1.766)
*N*	12,216	12,216	12,216
Adj. R^2^	0.107	0.181	0.184

*p < 0.1, **p < 0.05, ***p < 0.01. *T*-values are reported in parentheses, and these values are estimated using county-clustered heteroskedasticity-robust standard errors. All regression models control for county fixed effects, year fixed effects, and covariates related to older adults individuals, spouses, and households.

Column (4) demonstrates that the pilot policy did not significantly influence older adult individuals’ own perceptions of environmental quality. This may be because spouses, often serving as primary caregivers, are more attentive to local environmental conditions and thus more sensitive to environmental improvements.

In the Supplementary Appendix, we further explore the mediating effect of environmental quality using two objective city-level indicators: industrial sulfur dioxide emissions and soot emissions. We examined the strength of these mediating pathways through detailed analysis of the first-stage effect (Path a), and, drawing on existing literature, elaborated the mechanisms by which air pollution reduction improves older adults health. To ensure robustness, we replaced individual-level controls with city-level variables relevant to environmental conditions.

The analysis showed a significant reduction in pollutant emissions following the implementation of the low-carbon city pilot policy (see Supplementary Table A7). Prior studies have confirmed that improved air quality can lead to substantial health gains among older populations. These findings support the conclusion that the low-carbon pilot program has contributed to better older adults health outcomes by improving environmental quality, thus providing strong empirical support for Research Hypothesis 2.

Due to data limitations, this study does not include specific pollutant indicators such as PM2.5, NO₂, or SO₂. However, existing research shows that these pollutants affect older adults health through distinct mechanisms: PM2.5 is associated with cardiovascular and respiratory diseases (58); NO₂ is linked to reduced lung function and higher risk of chronic obstructive pulmonary disease (59); and SO₂ may trigger systemic inflammation and exacerbate health conditions (60). Therefore, it is reasonable to infer that the observed health improvements may be partly driven by reductions in these specific pollutants.

#### 4.2.5 Travel behavior mechanism

Table 7 reports the empirical results of the travel behavior mechanism. Column 1 shows that the low-carbon city pilot program significantly increased the use of older adults non-motorized travel. Column 2 indicates that this increase in non-motorized travel positively contributed to older adults health outcomes. Column 3 presents the results after controlling for older adults non-motorized travel. Although the coefficient of the policy variable slightly decreases compared to the baseline regression, it remains statistically significant. This suggests that part of the health improvement among the older adults was achieved through changes in travel behavior. These findings confirm that the low-carbon city pilot program promotes older adults health by encouraging the adoption of non-motorized travel modes, such as walking and cycling, thereby supporting Research Hypothesis 3.

**Table 7:** Mechanism of the low-carbon city pilot program’s impact on older adults health via non-motorized travel.

Variables	(1) Path a Older adults non-motorized travel	(2) Path b Older adults health	(3) Path c Older adults health
DID	0.070*** (2.745)		0.286* (1.898)
Older adults non-motorized travel		0.048* (1.941)	0.046* (1.812)
Controls	Yes	Yes	Yes
County FE	Yes	Yes	Yes
Year FE	Yes	Yes	Yes
*N*	12,646	12,646	12,646
Adj. R^2^	0.214	0.188	0.189

*p < 0.1, **p < 0.05, ***p < 0.01. *T*-values are reported in parentheses and estimated using robust standard errors clustered at the county level. All regressions include county fixed effects, year fixed effects, and control variables related to the older adults, their family members, and household characteristics.

### 4.3 Moderating effects

Table 8 examines how commuting distance and commuting time moderate the effect of the LCCP on older adults health outcomes. Columns 1 and 2 present the results based on commuting distance, while Columns 3 and 4 focus on commuting time. The DID coefficients indicate that the health effects of the pilot policy are statistically significant under conditions of longer commuting distances (Column 2) and longer commuting times (Column 4). This suggests that the health benefits of LCCPs become more pronounced for older adult individuals who face greater spatial constraints in their daily lives.

**Table 8:** Heterogeneity analysis by older adults commuting distance and time to activity centers.

Variables	(1) Short distance	(2) Long distance	(3) Short time	(4) Long time
DID	0.250 (1.615)	0.613*** (2.961)	0.305 (1.413)	0.301** (2.218)
Controls	Yes	Yes	Yes	Yes
County FE	Yes	Yes	Yes	Yes
Year FE	Yes	Yes	Yes	Yes
*N*	12,579	3,208	4,549	11,239
Adj. R^2^	0.189	0.162	0.192	0.174

*p < 0.1, **p < 0.05, ***p < 0.01. Robust *t*-statistics are reported in parentheses, clustered at the county level. All models include county fixed effects, year fixed effects, and individual-, parent-, and household-level control variables.

These results are further supported by robustness checks based on average commuting time groupings (see Supplementary Table A8). Across both indicators, the moderating role of spatial accessibility is evident: the further and longer older adult individuals must travel to reach essential activity centers, the greater the relative health improvement observed under the LCCP framework. This may be due to the fact that individuals facing longer travel burdens are more sensitive to the improvements in infrastructure, mobility, and environmental quality brought by LCCPs.

In line with Hypothesis 4, these findings confirm that commuting distance and time serve as significant moderators of the relationship between LCCPs and older adults health. Specifically, while all older adults individuals may benefit from policy interventions, those who previously faced longer distances or durations experience relatively greater gains once spatial and mobility-related constraints are eased. Conversely, individuals with already high accessibility may exhibit marginal improvements. Thus, spatial accessibility not only influences exposure to policy benefits but also shapes the extent of their realization.

### 4.4 Heterogeneity analysis

Table 9 presents the heterogeneous effects of the low-carbon city pilot policy on older adults health across subgroups defined by demographic characteristics. Specifically, Panels C, D, and E examine the differential impacts by gender, household registration (hukou) status, and age group.

**Table 9:** Heterogeneity analysis of older adults health outcomes.

Variables	Panel C Gender		Panel D Hukou status		Panel E Age group		
	Men	Women	Rural	Urban	Age 60–64	Age 65–69	Age 70–74
DID	0.362** (2.385)	0.231 (1.498)	0.488*** (2.655)	−0.047 (−0.294)	0.441*** (2.803)	0.328*** (1.952)	0.257** (1.800)
Controls	Yes	Yes	Yes	Yes	Yes	Yes	Yes
County FE	Yes	Yes	Yes	Yes	Yes	Yes	Yes
Year FE	Yes	Yes	Yes	Yes	Yes	Yes	Yes
*N*	8,248	7,545	12,546	3,245	2,599	8,263	4,895
Adj. R^2^	0.178	0.181	0.175	0.201	0.184	0.185	0.176

*p < 0.1, **p < 0.05, ***p < 0.01. *T*-values are reported in parentheses, estimated using robust standard errors clustered at the county level. All regressions control for county and year fixed effects, and covariates related to the son, spouse, and household.

Panel C shows that the policy significantly improved health outcomes among male older adults individuals. This gender difference may be attributed to variation in lifestyle and baseline health status. Male older adult individuals are generally more likely to engage in outdoor activities, making them more sensitive to improvements in air quality and travel environments. Additionally, they may have experienced poorer health conditions prior to the policy, providing greater potential for improvement. Men may also be more responsive to changes in travel infrastructure, thereby benefiting more from the policy’s promotion of low-carbon transportation.

Panel D reveals that the policy had a more pronounced effect among older adult individuals with rural hukou. This may be explained by the fact that rural areas have historically lagged behind urban areas in terms of air quality control, transportation infrastructure, and public service provision. As a result, the implementation of the low-carbon pilot policy may have brought about more concentrated environmental and infrastructural improvements in rural areas, leading to greater marginal health gains. Moreover, older adult residents in rural areas tend to rely more heavily on walking and cycling, increasing their sensitivity to changes in the local travel environment.

Panel E indicates that the policy produced stronger health benefits among the younger older adults group (aged 60–69). This finding likely reflects age-related differences in physical capacity and behavioral adaptability. Compared to older age groups, younger older adults individuals tend to have higher mobility and are more capable of adjusting their behavior in response to policy incentives—such as increasing non-motorized travel—which in turn promotes better health outcomes. In contrast, the oldest-old often face physical limitations that hinder their responsiveness to environmental changes, thereby reducing the effectiveness of the policy intervention.

In summary, the health impacts of the low-carbon city pilot policy vary across different older adult subgroups and are shaped by intersecting factors such as gender, household registration status, and age. These findings highlight the importance of considering demographic heterogeneity when evaluating public health outcomes of environmental policy.

## 5 Conclusion

Using panel data from the China Family Panel Studies (CFPS), this study applies a multi-period difference-in-differences (DID) approach to examine the impact of China’s Low-Carbon City Pilot Program (LCCP) on the health of older adults. The results show that the LCCP significantly improved self-rated health among individuals aged 60 to 75 and reduced the frequency of medical visits, confirming Hypothesis 1. These findings suggest that low-carbon urban development delivers not only environmental and economic benefits, but also significant social and health co-benefits.

Further mechanism analysis reveals that the policy enhanced perceived environmental quality among family members—particularly spouses—and that this perception is positively associated with older adults health outcomes (confirming Hypothesis 2). This indicates that the policy’s health effects are not only driven by objective air quality improvements but also rely on perception-based pathways. Based on this, local governments are encouraged to enhance the visibility and communication of environmental improvements—for example, by publishing street-level air quality data or encouraging community greening initiatives—to increase public recognition and behavioral responsiveness to environmental policies.

This study also shows that the policy operates through two main pathways. First, it significantly increased the share of older adults individuals using non-motorized transportation such as walking and cycling, forming a behavioral mechanism for health improvement (confirming Hypothesis 3). Based on this, local governments should invest more in age-friendly transport infrastructure, including barrier-free sidewalks, clear pedestrian signals, and bike lanes designed for older adults—especially in areas with high older adult populations. In addition, community activity centers and basic health services should be located within walking distance to encourage daily physical activity and reduce mobility-related health disparities.

Moreover, the results show that the policy has stronger health effects for older adults individuals with longer commuting distances and times, indicating that spatial accessibility plays a key moderating role (confirming Hypothesis 4). Therefore, cities should focus on improving service accessibility in peripheral or poorly connected areas, promote transit-oriented urban planning, and expand mobile healthcare and community service vans to lower transportation barriers to health benefits.

Heterogeneity analysis further reveals that the health benefits of the LCCP are more pronounced among men, rural residents, and the younger older adults (aged 60–69). These differences may result from varying baseline health conditions, behavioral responsiveness, and levels of environmental exposure across groups. Based on this, policymakers should take age and gender differences into account when designing low-carbon and health-related policies—for instance, by increasing investments in green energy and basic services in rural areas, or providing more in-home health support and indoor environmental improvements for the oldest older adults.

In sum, this study emphasizes that when climate-oriented urban policies are well-implemented and spatially inclusive, they can bring meaningful health co-benefits to aging populations. The findings offer important implications not only for China but also for other developing countries that are simultaneously facing the dual challenges of urbanization and population aging.

## Data Availability

The original contributions presented in the study are included in the article/Supplementary material, further inquiries can be directed to the corresponding author.
