# Supplementary material for: How do low-carbon city policies shape older adults health? Evidence from environmental and mobility pathways in China
Source: Front Public Health. 2025 Aug 20;13:1649879. doi: 10.3389/fpubh.2025.1649879 (PMC12405225; doi:10.3389/fpubh.2025.1649879)
Supplement: Supplementary file 1 [file Table_1.pdf]

Appendix A

TABLE A1 List of China's low-carbon city pilot program.

Pilot sets	Implementation dates	Provinces and cities included in China's low-carbon city pilot program
First set of pilot	July 19, 2010	Provinces: Guangdong, Liaoning, Hubei, Shaanxi, Yunnan; Cities: Tianjin, Chongqing, Shenzhen, Xiamen, Hangzhou, Nanchang, Guiyang, Baoding
Second set of pilot	November 26, 2012	Province: Hainan; Cities: Beijing, Shanghai, Shijiazhuang, Qinhuangdao, Jincheng, Hulun Buir, Jilin, Daxing'anling Prefecture, Suzhou, Huai'an, Zhenjiang, Ningbo, Wenzhou, Chizhou, Nanping, Jingdezhen, Ganzhou, Qingdao, Jiyuan, Wuhan, Guangzhou, Guilin, Guangyuan, Zunyi, Kunming, Yan'an, Jinchang, Urumqi
Third set of pilot	January 7, 2017	Cities: Wuhai, Shenyang, Dalian, Chaoyang, Xunke County, Nanjing, Changzhou, Jiaxing, Jinhua, Quzhou, Hefei, Huaibei, Huangshan, Lu'an, Xuancheng, Sanming, Gongqingcheng, Ji'an, Fuzhou, Jinan, Yantai, Weifang, Changyang Tujia Autonomous County, Changsha, Zhuzhou, Xiangtan, Chenzhou, Zhongshan, Liuzhou, Sanya, Qiongzhong, Chengdu, Yuxi, Simao District(Pu'er), Lhasa, Ankang, Lanzhou, Dunhuang, Xining, Yinchuan, Wuzhong, Changji, Yining, Hotan, Aral

Pilot announcement dates are based on official notices published by the National Development and Reform Commission (NDRC); When a province is designated as a pilot, all cities within its administrative boundary are included.

TABLE A2 Parallel trend test.

Variable	Coefficient	t-value
PRE2×TREAT	0.019	(0.11)
CURRENT×TREAT	0.348**	(2.18)
POST1×TREAT	0.174	(1.24)
POST2×TREAT	0.468***	(3.23)
Controls	Yes	Yes
County FE	Yes	Yes
Year FE	Yes	Yes
N	16,829	
Adj. R^2	0.26	

The initial period before policy implementation was designated as the reference group. PRE2 denotes two years prior to the policy implementation, CURRENT refers to the implementation year, POST1 indicates one year after implementation, and POST2 represents two years after implementation. *p < 0.1, **p < 0.05, ***p < 0.01. T-values are reported in parentheses and are estimated using robust standard

errors clustered at the county level. All regressions include county fixed effects, year fixed effects, and control variables related to the older adults individual, spouse, children, and household characteristics.

TABLE A3 Sub-sample analysis.

Variable	Eastern Region – Hebei	Central Region – Jiangxi	Western Region – Gansu
DID	1.195**<br>(2.251)	0.146***<br>(4.357)	0.861***<br>(4.544)
Controls	Yes	Yes	Yes
County FE	Yes	Yes	Yes
Year FE	Yes	Yes	Yes
N	1,086	609	2,250
Adj. R^2	0.261	0.330	0.231

*p < 0.1, **p < 0.05, ***p < 0.01. T-values are reported in parentheses and are based on robust standard errors clustered at the county level. All regressions include county fixed effects, year fixed effects, and control variables related to the spouse, children, and household characteristics.

TABLE A4 Covariate balance before and after propensity score matching (PSM).

Variable	Match Status	Treated Mean	Control Mean	% Bias	% Reduction in Bias	t-stat	p-value
Older adults age	Unmatched	9.5159	9.3692	4.4		2.73	0.01
	Matched	9.5159	9.4858	1.1	80.5	0.58	0.641
Older adults gender	Unmatched	0.60582	0.63141	-5.2		-3.24	0.003
	Matched	0.60582	0.60241	0.8	87.7	0.47	0.719
Urban residency (hukou)	Unmatched	0.36753	0.27015	23.8		14.96	<0.001
	Matched	0.36753	0.35798	2.4	90.3	1.29	0.334
Sleep duration	Unmatched	9.2489	9.2992	-5.2		-3.13	0.003
	Matched	9.2489	9.2343	1.5	71.0	0.89	0.439
Seeks medical care when ill	Unmatched	0.6899	0.74908	-12.3		-7.62	<0.001
	Matched	0.6899	0.69339	-0.8	94.2	-0.49	0.797
Living at home	Unmatched	0.9428	0.91515	7.5		4.57	<0.001
	Matched	0.9428	0.94297	0.0	99.5	-0.04	0.99
Spouse’s age	Unmatched	38.234	37.691	8.9		5.51	<0.001
	Matched	38.234	38.247	-0.3	97.7	-0.22	0.916
Son’s age	Unmatched	36.03	35.82	5.1		3.15	0.012
	Matched	36.03	36.182	-1.1	79.2	-0.68	0.574
Spouse’s education	Unmatched	3.161	2.9415	18.9		11.68	<0.001
	Matched	3.161	3.17	-0.9	96.0	-0.5	0.786
Son’s education	Unmatched	2.9932	2.6591	27.6		17.06	<0.001
	Matched	2.9932	2.9301	-2.3	92.0	-1.26	0.347
Spouse’s	Unmatched	7.3619	7.3605	0.0		0.02	0.995

smoking	Matched	7.3619	7.3627	0.0	40.5	0.00	0.998
Son's smoking	Unmatched	0.11585	0.08434	2.9		1.84	0.092
	Matched	0.11585	0.12208	-0.6	80.3	-0.37	0.889
Log family net income	Unmatched	10.785	10.595	16.1		9.98	<0.001
	Matched	10.785	10.764	1.8	89.4	0.98	0.343

After matching, the standardized mean differences (bias) for all covariates were reduced to below 3%, and all t-tests became statistically insignificant. These results indicate that the matched samples are well-balanced across observed characteristics, supporting the validity of the subsequent estimation using the Propensity Score Matching Difference-in-Differences (PSM-DID) method.

TABLE A5 Impact of the low-carbon city pilot program on the health of the older adults (PSM-DID Estimation).

**Variable**	**Coefficient**	**t-statistic**
DID	0.314**	(2.118)
Older adults age	0.007	(1.523)
Older adults gender	0.023	(0.797)
Urban residency (hukou)	0.115***	(2.739)
Sleep duration	−0.029*	(−1.723)
Seeks medical care when ill	−0.043	(−1.338)
Living at home	0.092**	(2.191)
Spouse's age	−0.017**	(−2.028)
Son's age	0.005	(1.170)
Spouse's education	0.044***	(3.056)
Son's education	0.085***	(6.399)
Spouse's smoking	0.015***	(4.022)
Son's smoking	−0.023	(−1.702)
Log family net income	0.054***	(3.850)
County FE	Yes	Yes
Year FE	Yes	Yes
N	15,927	
Adj. R^2	0.280	

*p < 0.1, **p < 0.05, ***p < 0.01. T-values are reported in parentheses and are based on robust standard errors clustered at the county level. The significantly positive coefficient of the DID variable confirms the robustness of the baseline regression results.

TABLE A6 Impact of low-carbon city pilot program on the health of the older adults using instrumental variable (IV) method.

**Variable**	**Topographic Relief**		**Urban Green Space Area**		**Both IVs**	
	1st Stage	2nd Stage	1st Stage	2nd Stage	1st Stage	2nd Stage
	DID	Older adults Health	DID	Older adults Health	DID	Older adults Health

DID (Treat × Post)		0.375** (2.288)		0.340** (2.429)		0.347** (2.509)
Topographic Relief × Post	0.797*** (4.633)				0.146*** (3.510)	
Urban Green Space Area × Post			0.201*** (30.821)		0.105*** (29.449)	
Under-identification test	Passed		Passed		Passed	
F-statistic	20.648		943.853		582.587	
Hansen J test					Passed	
Controls	Yes	Yes	Yes	Yes	Yes	Yes
County FE	Yes	Yes	Yes	Yes	Yes	Yes
Year FE	Yes	Yes	Yes	Yes	Yes	Yes
N	13,969	13,969	12,179	12,179	12,179	12,179
Adj. R^2	0.404	0.036	0.982	0.036	0.990	0.036

*p < 0.1, **p < 0.05, ***p < 0.01. T-values are reported in parentheses and are based on robust standard errors clustered at the county level. All regressions include control variables, county fixed effects, and year fixed effects. The under-identification test rejected the null hypothesis of weak instrument relevance at the 1% level. The F-statistics exceed the threshold of 10, indicating strong instruments. The Hansen J test suggests that topographic relief and urban green space area are valid instruments without over-identification concerns. The second-stage regression results confirm the positive effect of the low-carbon city pilot program on older adults health.

TABLE A7 The potential mechanism of the impact of low-carbon city pilot program on the health of the aged—by the improvement of environmental quality (Objective environmental quality indicators).

Variable	Industrial sulfur dioxide emissions	Industrial soot emissions
DID	-0.429*** (-3.327)	-0.460** (-2.607)
Per capita GRP	-0.429 (-0.675)	1.603 (1.344)
GRP growth rate	-0.330 (-1.435)	0.212 (0.874)
Population density	0.012*** (2.822)	0.003 (1.713)
Green area	-0.473 (-1.633)	0.426*** (2.948)
Proportion of the primary industry in GRP	1.999 (0.398)	17.377 (1.732)
Proportion of the secondary industry in GRP	1.966 (0.391)	17.281 (1.726)
Proportion of the tertiary industry in GRP	1.931 (0.386)	17.260 (1.724)
Number of industrial enterprises above a certain size	-0.000 (-1.446)	-0.000** (-2.115)
Total industrial output value above a certain size	1.135 (1.725)	0.667* (1.913)

County FE	Yes	Yes
Year FE	Yes	Yes
N	6,728	6,757
Adj. R^2	0.973	0.948

*p < 0.1, **p < 0.05, ***p < 0.01. GRP referred to the Gross Regional Product. T-values were reported in parentheses, which were estimated using the robust standard errors of cluster heteroscedasticity at the county level. Both regressions controlled for county FE and year FE. The control variables were per capita GRP, GRP growth rate, population density, green area, the proportion of primary, secondary, and tertiary industries in GRP, the number of industrial enterprises above a certain size, and the total output value of industrial enterprises above a certain size. Both the objective environmental quality indicators and the control variables were converted to logarithmic form as the underlying data were skewed. Data pertaining to industrial sulfur dioxide emissions, industrial soot emissions and control variables were extracted from China's Urban Statistical Yearbooks.

TABLE A8 Heterogeneity analysis by commuting time among the older adults.

Variable	Short commuting time	Long commuting time
DID	0.385	0.318*
	(1.688)	(1.896)
Controls	Yes	Yes
County FE	Yes	Yes
Year FE	Yes	Yes
N	8,625	7,355
Adj. R^2	0.284	0.270

The average commuting time of 14 minutes serves as the threshold, with individuals below this point categorized as having a "short commuting time to activity center", and those above it categorized as having a "long commuting time to activity center". *p < 0.1, **p < 0.05, ***p < 0.01. T-values were reported in parentheses, which were estimated using the robust standard errors of cluster heteroscedasticity at the county level. Both regressions controlled for county FE, year FE and control variables related to the spouse, children, and family.
